# Symbolic Representation and Learning With Hyperdimensional Computing

**DOI:** 10.3389/frobt.2020.00063

**Published:** 2020-06-09

**Authors:** Anton Mitrokhin, Peter Sutor, Douglas Summers-Stay, Cornelia Fermüller, Yiannis Aloimonos

**Affiliations:** ^1^Computer Vision Laboratory, Department of Computer Science, University of Maryland Institute for Advanced Computer Studies, University of Maryland, College Park, MD, United States; ^2^Computational and Information Sciences Directorate, Army Research Laboratory, Adelphi, MD, United States

**Keywords:** hyperdimensional computing, semantic vectors, hashing, machine learning, image processing

## Abstract

It has been proposed that machine learning techniques can benefit from symbolic representations and reasoning systems. We describe a method in which the two can be combined in a natural and direct way by use of hyperdimensional vectors and hyperdimensional computing. By using hashing neural networks to produce binary vector representations of images, we show how hyperdimensional vectors can be constructed such that vector-symbolic inference arises naturally out of their output. We design the Hyperdimensional Inference Layer (HIL) to facilitate this process and evaluate its performance compared to baseline hashing networks. In addition to this, we show that separate network outputs can directly be fused at the vector symbolic level within HILs to improve performance and robustness of the overall model. Furthermore, to the best of our knowledge, this is the first instance in which meaningful hyperdimensional representations of images are created on real data, while still maintaining hyperdimensionality.

## 1. Introduction

Over the past decade, Machine Learning (ML) has made great strides in its capabilities to the point that many today cannot imagine solving complex, data-hungry tasks without its use. Indeed, as learning by example is a very necessary skill for an artificial general intelligence, it seems that ML's success bodes its necessity - in some form or other - in future AI systems. At the same time, end-to-end ML solutions suffer from several disadvantages; results are generally not interpretable or explainable from a human perspective, new data is difficult to absorb without significant retraining, and the amount of data/internalized knowledge required to train can be untenable for tasks that are easy for humans to solve. Symbolic reasoning solutions, on the other hand, can offer a solution to these problems.

One issue with symbolic reasoning is that symbols preferred by humans may not be easy to teach an AI to understand in human-like terms. Problems like these have led to the interesting solution of representing symbolic information as vectors embedded into high dimensional spaces, such as systems like word2vec (Mikolov et al., 2013) or GloVe (Pennington et al., 2014). These are often used to inform other symbolic or ML systems to give semantic context to information represented textually. In some systems, symbolic concepts themselves are represented entirely as high dimensional vectors that coexist in a common space-these are often referred to as Vector Symbolic Architectures (VSA). This notion is of particular interest, as many ML techniques produce such high dimensional vectors as a byproduct of their learning process or their operation.

In this article, we have focused on the notion of combining ML systems and VSA using high dimensional vectors directly. Specifically, we focused on the use of hyperdimensional vectors and Hyperdimensional Computing to achieve this (Kanerva, 2009). The properties of hyperdimensionality give rise to interesting ways to manipulate symbolic information so long as that information can be represented with long binary vectors. Moreover, this combination is achieved naturally, and is highly modifiable. Hyperdimensional computing can even improve the results of ML methods. Since hyperdimensional computing requires a method to convert data into long binary vectors, we focused mostly on hashing techniques for images, though the results are applicable for any ML approach that produces long binary vectors, either by directly producing them or by a special encoding. This allows a convenient method for converting images into hyperdimensional representations that naturally work with symbolic reasoning systems, such as fuzzy logic systems.

**Figure 1:**
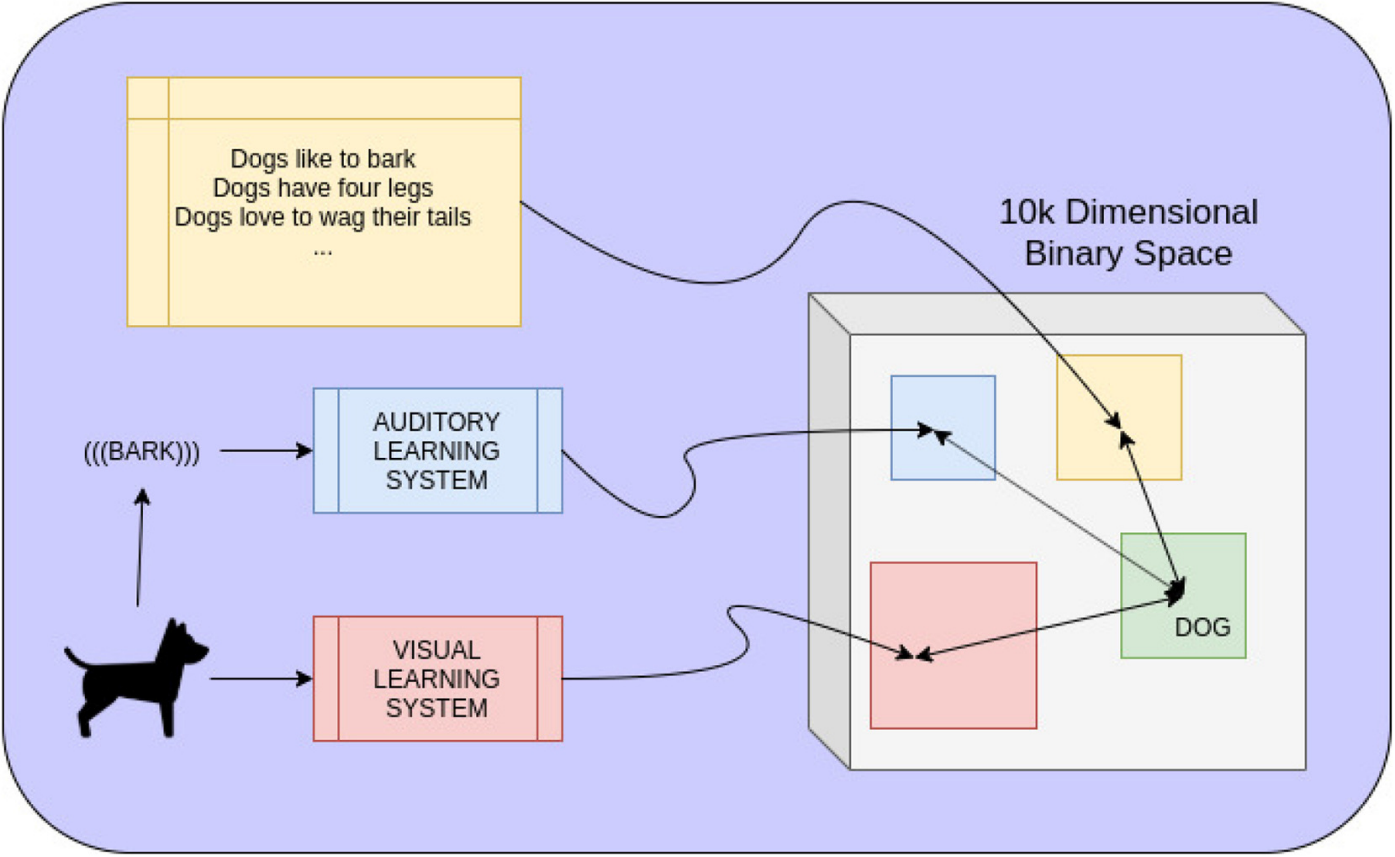
Mapping different modalities of information to the same space of long binary vectors allows knowledge of the world to coexist and combine together symbolically as well. A dog may be seen and heard, recognized by two separate data-driven learning systems. The output of each, representing the presence of a dog, is mapped to a binary vector representing the current data. The closer this mapping is to a learned representation of all dogs, the more likely it is to be a dog. In the same space, linguistic knowledge of dogs can also be mapped to symbolic representations. Combining all three modalities by purely hyperdimensional computations gives a single symbolic representation of everything pertaining to the concept of dogs.

Consider Figure 1, which demonstrates how hyperdimensional vectors could be used to convert data driven systems of different modalities to a shared space of long binary vectors. Once mapped to such a space, where distance between mappings is meaningful, it is clear that the binary space is a purely symbolic representation of both the input to each data-driven system and their respective output. Once symbolically represented, operations performed in the hyperdimensional space can further map vectors to more complicated representations: in this particular instance, the symbolic concept of a dog. In a more complicated system, the entire hyperdimensional space can be overlayed by a knowledge graph, fuzzy logic system, VSA, or any other symbolic reasoning system. We largely focused on how to achieve this mapping from an external learning system to a binary space and, consequently, how to symbolically “fuse” different modalities together to get a better symbolic representation of a real-world, data-driven concept. Our experimental results indicate impressive improvement in terms of performance, when fusing the outputs of multiple data-driven models, at little to no computational cost. The structure of this fusing allows for more models to be added or removed as desired without requiring expensive computation or retraining.

The remainder of this article is structured as follows. First, in section 2, we have provided necessary background information on hyperdimensional computation. Next, in section 3, we have discussed related work and results that are pertinent to this article. Then, in section 4, we have presented the architecture of a system that could achieve the desired functionality shown in Figure 1 and shown how it can be trained and used at testing time. Of particular importance is the notion of the Hyperdimensional Inference Layer, which can effectively fuse symbolic representations in the hyperdimensional space. In section 5, we have outlined an experiment to test how well such an architecture would work in practice. Notably, we have constrained ourselves to using image hashing networks and have shown that not only can our architecture effectively fuse the outputs of different networks together in the hyperdimensional space but also that the mere usage of hyperdimensional vectors as a memory mechanism can improve performance as well. Naturally, in section 6, we have shown the results of these experiments. Finally, in section 7, we have discussed our results and outlined the pros/cons of using hyperdimensional vectors to fuse learning systems together at a symbolic level as well as what future work is necessary.

## 2. Background Information

We first covered some of the relevant properties of hyperdimensional vectors for comprehension, as discussed in Kanerva (2009). Hyperdimensionality arises in binary vectors of sufficiently long length, usually on the order of 10,000 bits. Given two random vectors *a* and *b* from *B*^*n*^ = {0, 1}^*n*^ for large *n*, their overlap in bits has a high probability of being close to the expected value of *n*/2 with a standard deviation of $\sqrt{n/4}$. Therefore, two randomly selected vectors will overwhelmingly have a Hamming Distance of *n*/2; in this case, we can say the vectors are uncorrelated.

Two vectors *a* and *b* can be bound together by using the *exclusive-or* (XOR) operation, which we will represent with * symbol:

(1) $$\begin{array}{r} c=a*b \end{array}$$

Trivially, given one of the vectors, say *a*, we can unbind *c* to get *b*:

(2) $$\begin{array}{r} a*c=a*\left(a*b\right)=\left(a*a\right)*b=b \end{array}$$

Suppose that *a* and *b* represent symbolic concepts; binding them with Equation (1) and unbinding with Equation (2) produces a new symbolic concept *c* that is deconstructed to its atomic symbols. Additionally, it is trivial that the Hamming Distance between two vectors is preserved when both are mapped by either another XOR with *m* or by a common permutation Π of bits:

(3) $$\begin{array}{r} \left|a_{m}*b_{m}|=|\left(a*m\right)*\left(b*m\right)|=|a*b\right| \end{array}$$

(4) $$\begin{array}{r} \left|\Pi a*\Pi b|=|\Pi \left(a*b\right)|=|a*b\right| \end{array}$$

For our purposes, permutation and XOR are used interchangeably as “multiplication” operations. In order to create a more sophisticated and structured vector, we required an “addition” operation. We primarily concerned ourselves with the “consensus sum,” where each bit of the resultant vector is set to be the bit value that appears more often in that component across the terms:

(5) $$\begin{array}{r} {+}_{c}\left(\left\{a_{1},a_{2},\ldots ,a_{l}\right\}\right)=a_{1}{+}_{c}a\ldots {+}_{c}a_{l}=a_{c} \end{array}$$

where for a count *z* of 0's across the *l* terms:

(6) aci={0 & z > l/2 1 & z < l/2 random & z = l

However, if permutation is used for multiplication, it is valid to use XOR for addition. For either * or +_*c*_ as the + operator, a sequence of symbolic information *A* = *A*_1_*A*_2_…*A*_*l*_ can be represented as

(7) $$\begin{array}{r} \begin{array}{c} a=\Pi \left(\ldots \left(\Pi \left(\Pi a_{1}+a_{2}\right)+a_{3}\right)+\ldots \right)+a_{l}\\ =\Pi^{l-1}a_{1}+\Pi^{l-2}a_{2}+\ldots +\Pi a_{l-1}+a_{l} \end{array} \end{array}$$

where *a*_*i*_ are vector representations of corresponding *A*_*i*_ and Π is a permutation that represents the sequence. When using XOR, subsquences can be removed, replaced, or extended by constructing them and XOR-ing with *a*.

Finally, a *record* *r* of fields *f* = [*f*_1_, *f*_2_, = …, *f*_*l*_], and their values *v* = [*v*_1_, *v*_2_, …, *v*_*l*_] can be constructed symbolically by binding each *f*_*i*_ with its corresponding *v*_*i*_ using Equation (1) and summing the result with Equation (5):

(8) $$\begin{array}{r} r=v\cdot f=v_{1}*f_{1}{+}_{c}\ldots {+}_{c}v_{i}*f_{i}{+}_{c}v_{l}*f_{l} \end{array}$$

Given a value *v*_*x*_, a record *r* can be probed by performing an XOR and finding the *f*_*j*_ with the smallest Hamming Distance, thus checking the existence of a field:

(9) $$\begin{array}{r} \underset{j}{\min}|\left(v_{x}*r\right)*f_{j}| \end{array}$$

A similar approach can be done to approximately recover the value of a field:

(10) $$\begin{array}{r} \underset{j}{\min}|\left(f_{x}*r\right)*v_{j}| \end{array}$$

When the bits of a probe do not correlate with a term in *r*, the term collapses into a noise vector, whereas the term that does correlate will produce a signal that approximately undoes the XOR binding.

## 3. Related Work

**Figure 2:**
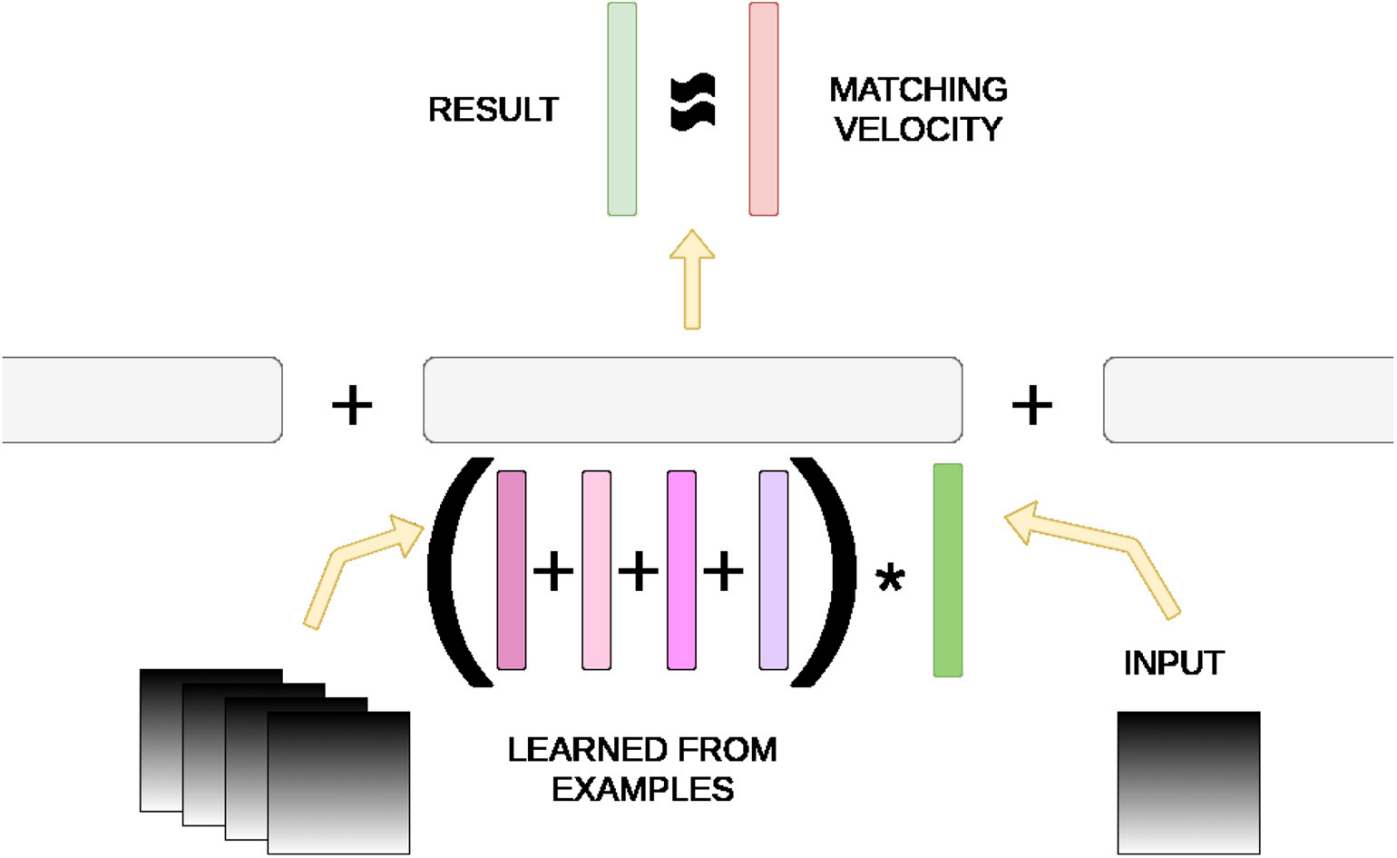
Hyperdimensional memory mechanism in HAP (Mitrokhin et al., 2019). A memory unit *m* consists of velocity bins as fields that are bound to another record of summed vector representations of time image slices from training. An input image is converted to its hyperdimensional vector representation and XOR'd with *m*. If it matches approximately with one of the image slice sums, the result contains the matching velocity representation with some noise.

Our work is primarily an extension of HAP (Mitrokhin et al., 2019) in which drones are trained to predict their egomotion in 3-D space. A neuromorphic camera's events are converted into a time image slice of motion, represented as a sparse RGB image, whose pixel data is symbolically represented with a structured hyperdimensional vector. Raw RGB values are semantically embedded into the hyperdimensional space such that each color component is closer to its nearest values than further values. Possible velocity values are finely binned and likewise semantically embedded. A structured record *m* is constructed to associate the egomotion in one component velocity to another record containing all time image slices that fall into the same velocity bin, with Equation (8), at training time. Egomotion prediction is achieved purely by these memory units *m* via XORing a novel time slice image with *m* and probing the possible velocities to find the closest match. Figure 2 demonstrates this process.

This method works because of the sparseness of pixel data in time image slices. The collection of time slices that are associated to a velocity bin average out to be representative of the motion changes the neuromorphic camera experienced. Surprisingly, this is sufficient to achieve neural-network-like performance, with a tiny fraction of memory, training samples, computation power, and training time of a neural approach. However, it is completely interpretable, can be trained online. and is effectively a symbolic reasoning system. Unfortunately, regular image data is too dense in information for this approach to work as implemented in HAP (Mitrokhin et al., 2019).

There exist other methods that have used hyperdimensional techniques to perform recognition (Imani et al., 2017) and classification (Moon et al., 2013; Rahimi et al., 2016; Imani et al., 2018; Kleyko et al., 2018). As with HAP (Mitrokhin et al., 2019), there have been other attempts to perform feature and decision fusion (Jimenez et al., 1999) or paradigms that can operate with minuscule amounts of resources (Rahimi et al., 2017). We differ from these approaches in that we try to assume as little about the model as possible except that it would be used in some form of classification for information that can be represented symbolically and modified with additional classifiers. Our results are a benchmark to see how much a hyperdimensional approach could facilitate a direct connection between ML systems and symbolic reasoning. On the solely symbolic representation and reasoning side, there exists relevant work on using cellular automata based hyperdimensional computing (Yilmaz, 2015). Some formulations based on real-valued vectors can also exhibit similar properties to long binary vectors so far as compositionality and decompositionality is concerned (Summers-Stay et al., 2018).

## 4. Architecture

Extending the model from HAP (Mitrokhin et al., 2019), the input vector is treated as any output from an ML system and the output velocity bins are now a symbolic representation of the output classes of the network. These would then feed in to a larger VSA system, that could feasibly be composed of other ML systems. Suppose that we have a pre-trained ML system, such as a Hashing Network, which can produce binary vectors as output to represent images.

### 4.1. Training the Hyperdimensional Inference Layer

**Figure 3:**
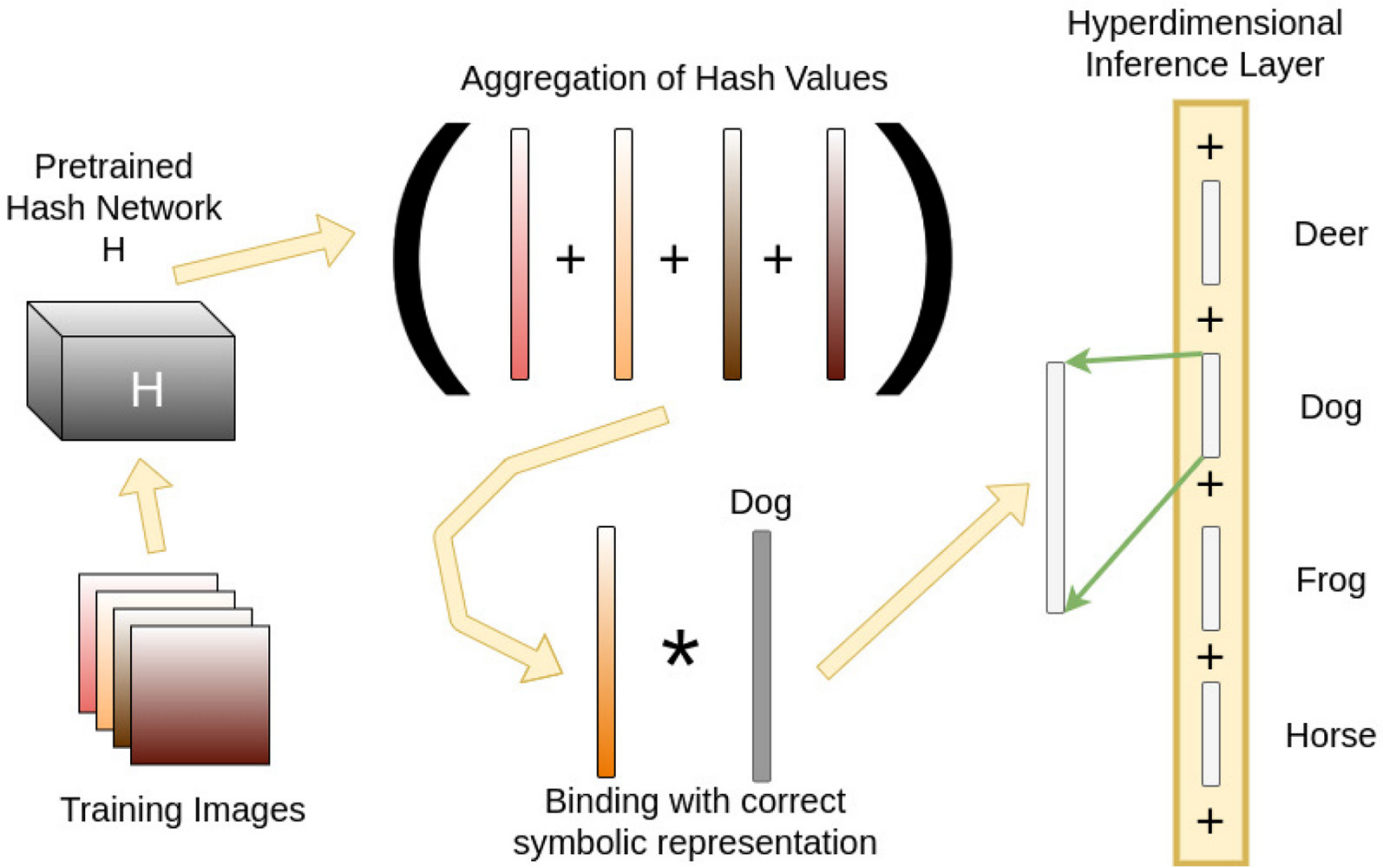
The training pipeline for a particular class of “dog.” First, training images are hashed into binary vectors using a pre-trained network. The vectors for each image are then projected to a hyperdimensional length by randomly repeating the bits consistently. Each vector is aggregated by the consensus sum operation into a single vector containing all training instances for that class. A symbolic representation of the class, called “Dog” in this example, as another hyperdimensional vector, is bound to the aggregated vector. This forms the association between representative images and the class itself. Once these inference vectors are computed for each class, they are aggregated by consensus sum into the Hyperdimensional Inference Layer, which then performs classification at testing time.

For a classification task, during training time, training images are hashed into binary vector representations. These are aggregated with the consensus sum operation in Equation (5) across their corresponding gold-standard classes, and a random basis vector meant to symbolically represent the correct class is bound to the aggregate with Equation (1). The resultant vector now represents a *memory* containing all training instances observed but that are represented symbolically with appropriated hashed binary vectors that are projected into a hyperdimensional binary space by randomly permuting and assembling the hash vector. Figure 3 shows this process when training to classify a “dog” in an image. This dog class is aggregated into a larger vector, once again with the consensus sum operation in Equation (5), to produce a hyperdimensional vector containing similar memory vectors across the other classes. This is referred to as the Hyperdimensional Inference Layer (HIL), which then infers the correct class at testing time for a novel image.

### 4.2. Testing the Hyperdimensional Inference Layer

**Figure 4:**
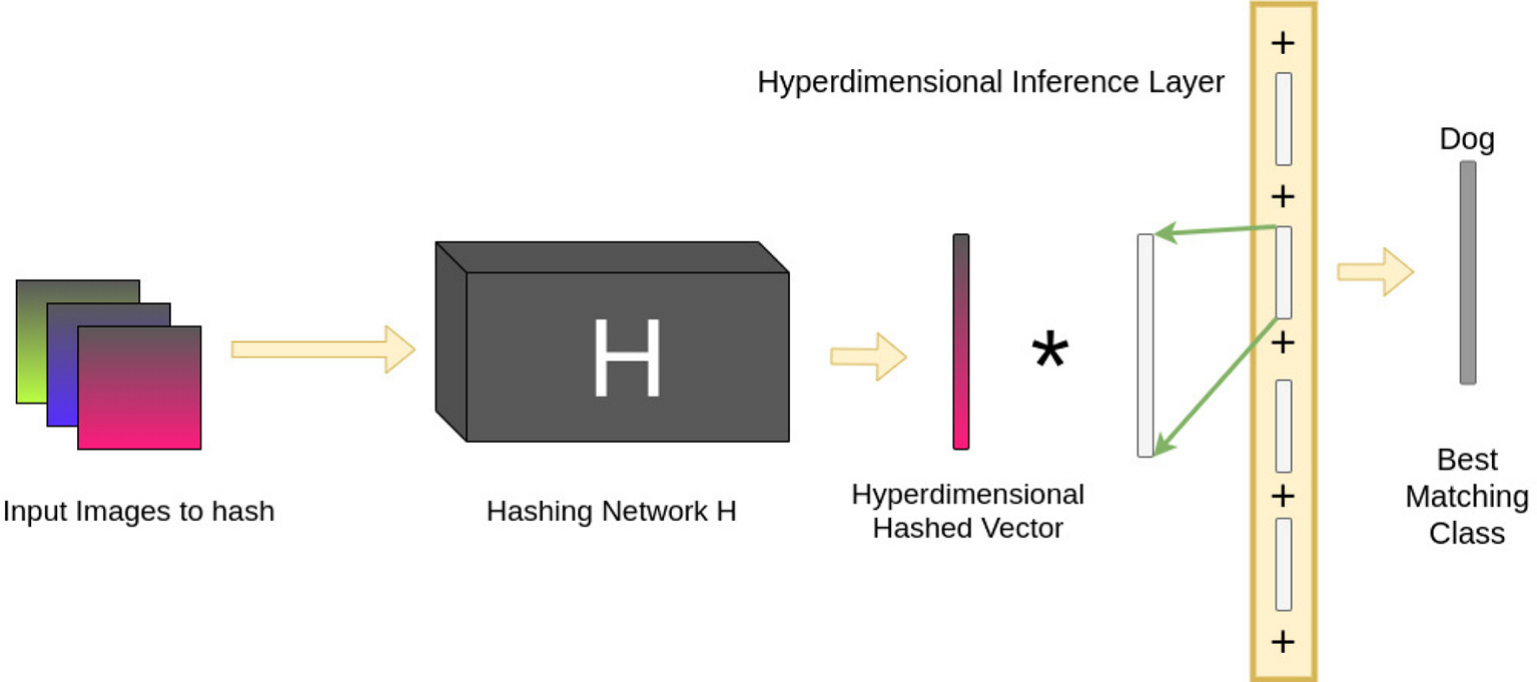
The pipeline for testing the Hyperdimensional Inference Layer. Images are presented to the hashing network *H*, which are then hashed into binary vectors. As during training, these are projected into hyperdimensional vectors. The result is then XOR'd with the HIL. The XOR distributes across the terms in the HIL and creates noise for terms corresponding to incorrect classes. Only the correct class will deviate from the noise and will be detected as the best matching class by computing the Hamming Distance between the result of the XOR and every vector representation of the classes; the class with the smallest Hamming Distance is selected as the correct classification.

Once training is complete, classification of a novel image is relatively straightforward. An image is converted to a binary vector by the pre-trained hashing network. This vector is then projected into a hyperdimensional vector in the same manner as during training. Finally, the XOR between this vector and the HIL is computed. The Hamming Distance between the resultant vector and each of the class representations is measured. The class vector with the smallest Hamming Distance is selected as the correct classification. Figure 4 shows this process in action.

### 4.3. Consensus With Multiple Models

**Figure 5:**
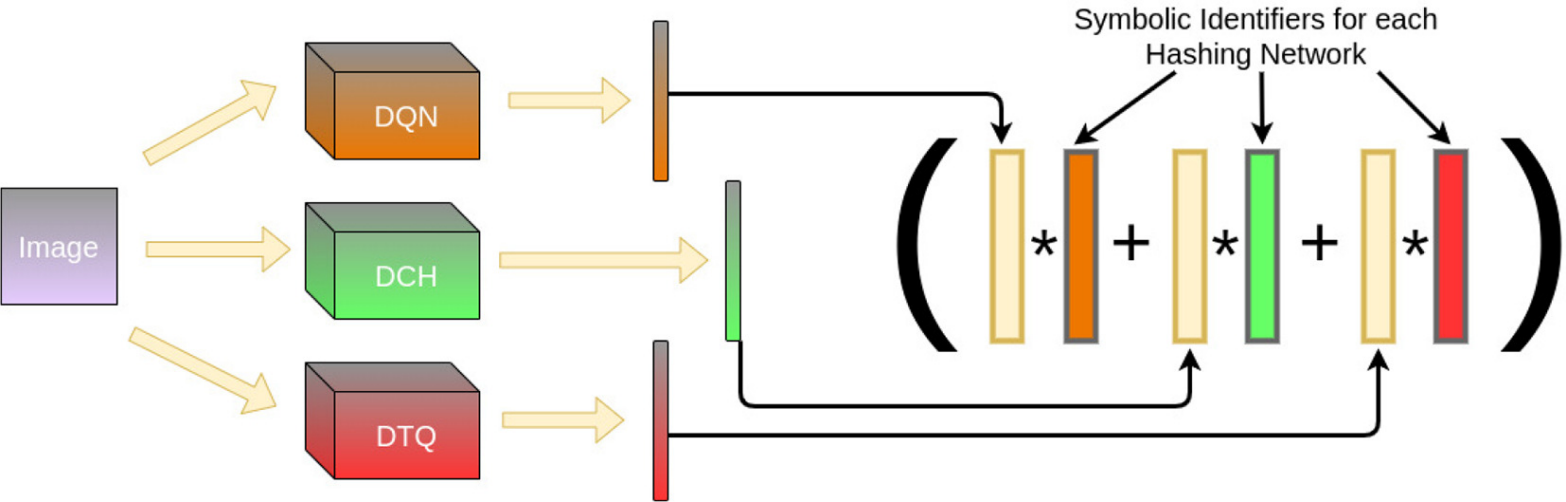
Given multiple ML models, the HIL of each can be fused together by repeating the same training procedure. Thus, given an image, each hashing network converts it to a different binary vector, which is projected into hyperdimensional lengths. These are bound with symbolic vectors identifying each individual hashing network and aggregated via consensus sum. The result allows us to perform inference across multiple models at testing time.

One advantage of the hyperdimensional architecture for inference is how it can be easily manipulated. Of particular interest is when there are multiple models that can produce features in the form of hyperdimensional vectors for an input. Suppose we had several models, each with their own advantages. We can fuse their output together to form a consensus system that will consider each network's feature output before classification. We simply repeat the same method as we did for our classes but with symbolic identifiers for which model aggregated which data. Prediction is done as before, probing each model's output with XOR and finding the closest matching network vector. Figure 5 demonstrates how this pipeline would work.

## 5. Materials and Methods

The methodology, external systems, and datasets used for testing were as follows.

### 5.1. Methodology

To test how well hyperdimensional vectors can facilitate the mapping from the input/output of an ML system to a symbolic system, we required a model problem where it was possible to convert an ML result into hyperdimensional vectors. We studied the typical image classification problem but with hashing networks, as they directly convert raw images into binary vectors of variable length, which are used for classification and ranking based on Hamming Distance. This is simply done for convenience, as most neural methods do not product binary vectors of such large length that are also rankable, and we did not want other methods for embedding real numbered vectors into binary spaces to affect the results. We utilized the *DeepHash*^1^ library, which incorporates recent deep hashing techniques for image classification and ranking (Cao et al., 2016, 2017, 2018; Zhu et al., 2016; Liu et al., 2018). Our goal wsa to show that an added layer of inference to the outputs of these methods with hyperdimensional computing allows us to convert their results into common length, hyperdimensional vectors, without losing performance. In fact, as we have seen, performance can even increase.

Two separate experiments were performed to evaluate how well a structure like the one shown in Figure 1 would work in practice. Again, we limited ourselves to visual learning systems for simplicity, though there is no reason for such a limitation in practice.

1. We first tested how well a hyperdimensional representation of a given hashing network's output can work with a HIL. That is, does the inclusion of a HIL (and by extension, hyperdimensional representations) obfuscate the classification, thereby worsening performance, or does it perhaps improve the performance? In theory, the system should not do worse. However, the nature of HIL's structure may enable a better *memorization* of training examples. We trained individual Hash Networks to perform image classification and then compared their performance with and without a HIL. Performance is tracked by comparing the F1 score for classification to the number of training iterations for the Hash network or epochs. We were also interested in how the HIL affects the F1 score as the Hamming Distance threshold for similarity increases.
2. Additionally, we studied whether the HIL could improve the overall performance of our Hash Networks if we fused them at the symbolic level of their outputs, using a HIL, as shown in Figure 5. We designed an experiment where all networks used in our first experiment are combined together by fusing their individual HIL into a new HIL. The idea asiws that, individually, these Hash Networks have different strengths and weaknesses based on their formulation. When fused into a HIL, each contributes toward the overall classification result, allowing the best matching classification across all models simultaneously.

### 5.2. External Systems

We used three of the image hashing networks from DeepHash in our experiments. In the following sections, we have described and outlined each one individually. In general, these networks use features provided by another system and compute hashes based on features extracted from the images into compact codes for image retrieval and classification. Additionally, we built our hyperdimensional inference layer by using the open source framework pyhdc^2^ library, as used in HAP (Mitrokhin et al., 2019), which contains basic, but very efficiently implemented, operations for hyperdimensional computing and representing hyperdimensional vectors. Finally, AlexNet (Krizhevsky et al., 2012) features pre-trained on ImageNet (Deng et al., 2009) are used in the DeepHash pipeline and are available for download from the GitHub repository.

#### 5.2.1. Deep Quantization Network (DQN)

The *Deep Quantization Network* (DQN) is a hashing-by-quantization network used for efficient image retrieval (Cao et al., 2016). The system supervises its hashing and allows statistical minimization of quantization errors from hand-crafted or machine learned features in a step separate from what traditional quantized hashing networks did prior. DQN formally controls this quantization error. The system is composed of four main subsystems:

1. Multiple convolution-pooling layers that capture deep image representations.
2. A fully connected layer that bottlenecks deep representations and projects them into an optimal lower dimensional representation for hashing.
3. A pairwise cosine layer for learning similarity preservation.
4. The quantization loss product that controls the quality of the hash and quantizes the bottleneck representations.

#### 5.2.2. Deep Cauchy Hashing Network (DCH)

The *Deep Cauchy Hashing Network* (DCH) seeks to improve hash quality by penalizing similar image pairs having a Hamming Distance bigger than the radius specified by the hashing network (Cao et al., 2018). The authors argue that hashing networks tend to concentrate related images within a specified Hamming ball due to mis-specified loss function. By penalizing the network for when this happens with a pairwise cross-entropy loss based on a Cauchy distribution, the rankings become stronger.

#### 5.2.3. Deep Triplet Quantization Network (DTQ)

The *Deep Triplet Quantization Network* (DTQ) further improves hashing quality by incorporating similarity triplets into the learning pipeline. By a new triplet selection approach, Group Hard, triplets are selected randomly from each image group that are deemed to be “hard.” Binary codes are further compacted by use of triplet quantization with weak orthogonality at training time.

### 5.3. Datasets

Evaluations of the hashing networks by themselves and with the hyperdimensional inference layer are performed on the CIFAR-10 standard dataset (Krizhevsky and Hinton, 2009) and the NUSWIDE_81 dataset (Chua et al., 2009), which contains tagged Flickr images with 81 concepts for classification.

## 6. Results

In the following sections, we present the results of our evaluation of the hyperdimensional inference layers in both experiments.

### 6.1. Hyperdimensional Inference Layer Results

To test the capabilities of the hyperdimensional inference layers in preserving the output of ML models when transformed into hyperdimensional vectors, we compared the performance of each hashing network individually vs. the performance when the hyperdimensional inference layer is added to the hashing network, as shown in Figures 3, 4.

#### 6.1.1. Results for CIFAR-10

**Figure 6:**
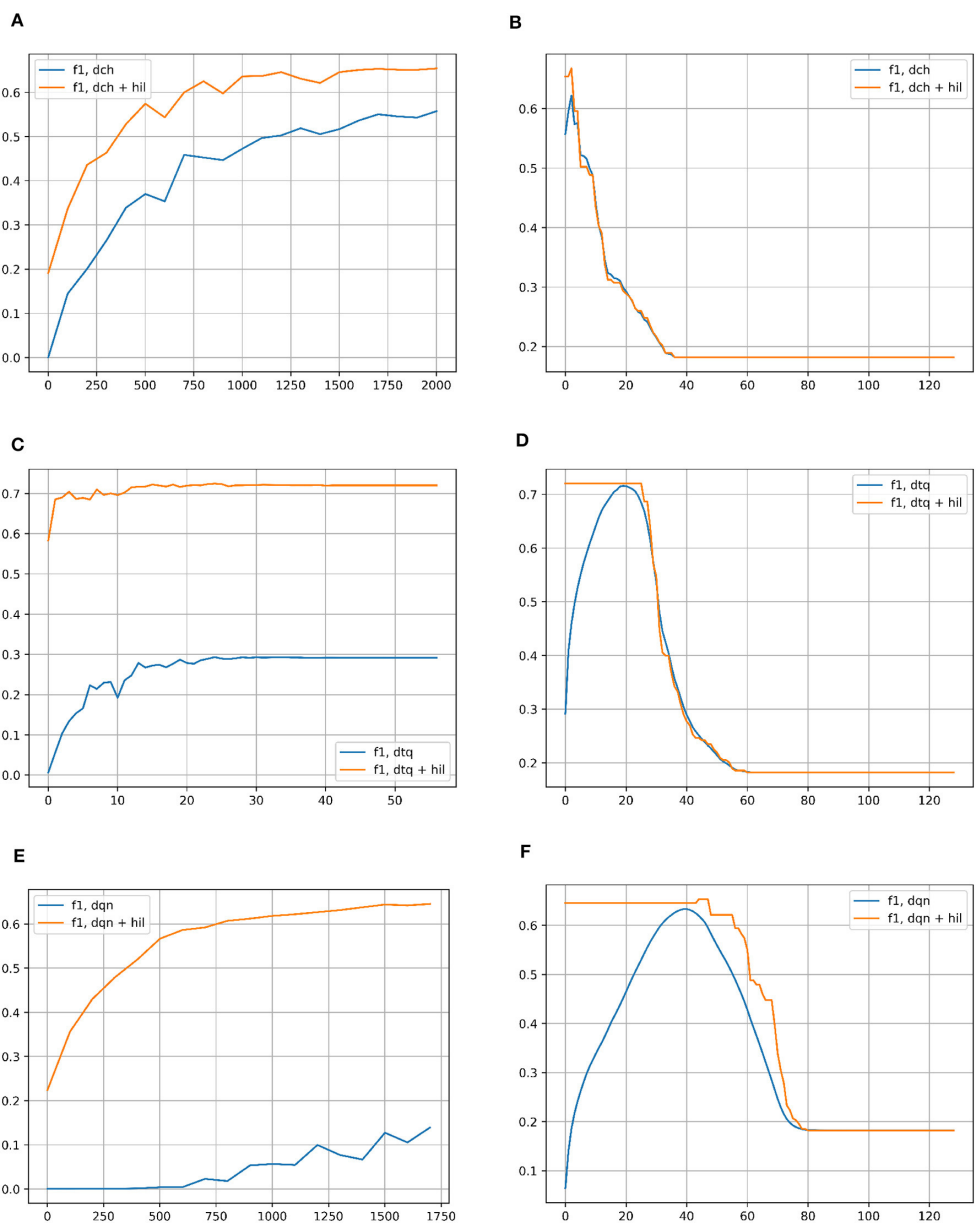
**(A)** F1 score for classification on the CIFAR-10 dataset with DCH with and without the HIL, as a function of the number of iterations of training of the DCH network. **(B)** F1 score for classification on the CIFAR-10 dataset with DCH with and without the HIL, as a function of the Hamming Distance for classification. The networks are fully trained to the end point shown in subplot **(A)**. **(C)** F1 score for classification on the CIFAR-10 dataset with DTQ with and without the HIL, as a function of the number of iterations of training of the DTQ network. **(D)** F1 score for classification on the CIFAR-10 dataset with DTQ with and without the HIL, as a function of the Hamming Distance for classification. The networks are fully trained to the end point shown in subplot **(C)**. **(E)** F1 score for classification on the CIFAR-10 dataset with DQN with and without the HIL, as a function of the number of iterations of training of the DQN network. **(F)** F1 score for classification on the CIFAR-10 dataset with DQN with and without the HIL, as a function of the Hamming Distance for classification. The networks are fully trained to the end point shown in subplot **(E)**. Baseline networks are shown in blue, while the same network with a HIL appended to the end is shown in yellow. Note that in the left column of subplots, the Hamming Distance for classification is set to 2 for inlier/outlier count. The left column of results show that the HIL boosts the speed at which the network trains, achieving a higher performance in far fewer iterations of expensive network training. As the HIL adds negligible overhead in memory/computation time, there is no downside to using a HIL. The right column of results show that the HIL prevents the need for searching for an optimal Hamming Distance threshold to classify with, as it supercedes peak performance of the network right away for the lowest possible distance thresholds. After peak performance of the baseline network, larger Hamming Distance thresholds eventually decay to the performance of the baseline.

Figure 6 compares the F1 scores of each hashing network with and without the HIL on CIFAR-10. The left column shows performance across iterations of network training (DTQ shows epochs instead). The threshold for Hamming Distance to search in is set to 2 (out of 128 bit vectors) for the baseline networks. For HIL results, as the vectors are hyperdimensional, the threshold is set to be proportionally that many bits out of 8,000. In all cases, the HIL improves performance greatly and with less iterations/epochs. In the right column, the F1 score is shown for successively more lax Hamming Distances in both methods, taking the best matching vector in a Hamming ball of that size. In the case of hyperdimensional vectors for the HIL, the size is once again proportional to 8,000 bit long vectors. For each baseline hashing network, there is clearly an optimal Hamming Distance to use. This is not the case for HIL, where it plateaus in each case for any distance smaller than the peak. As the number of bits increase, the performance quickly degrades to be more in line with the hashing network.

#### 6.1.2. Results for NUSWIDE-81

**Figure 7:**
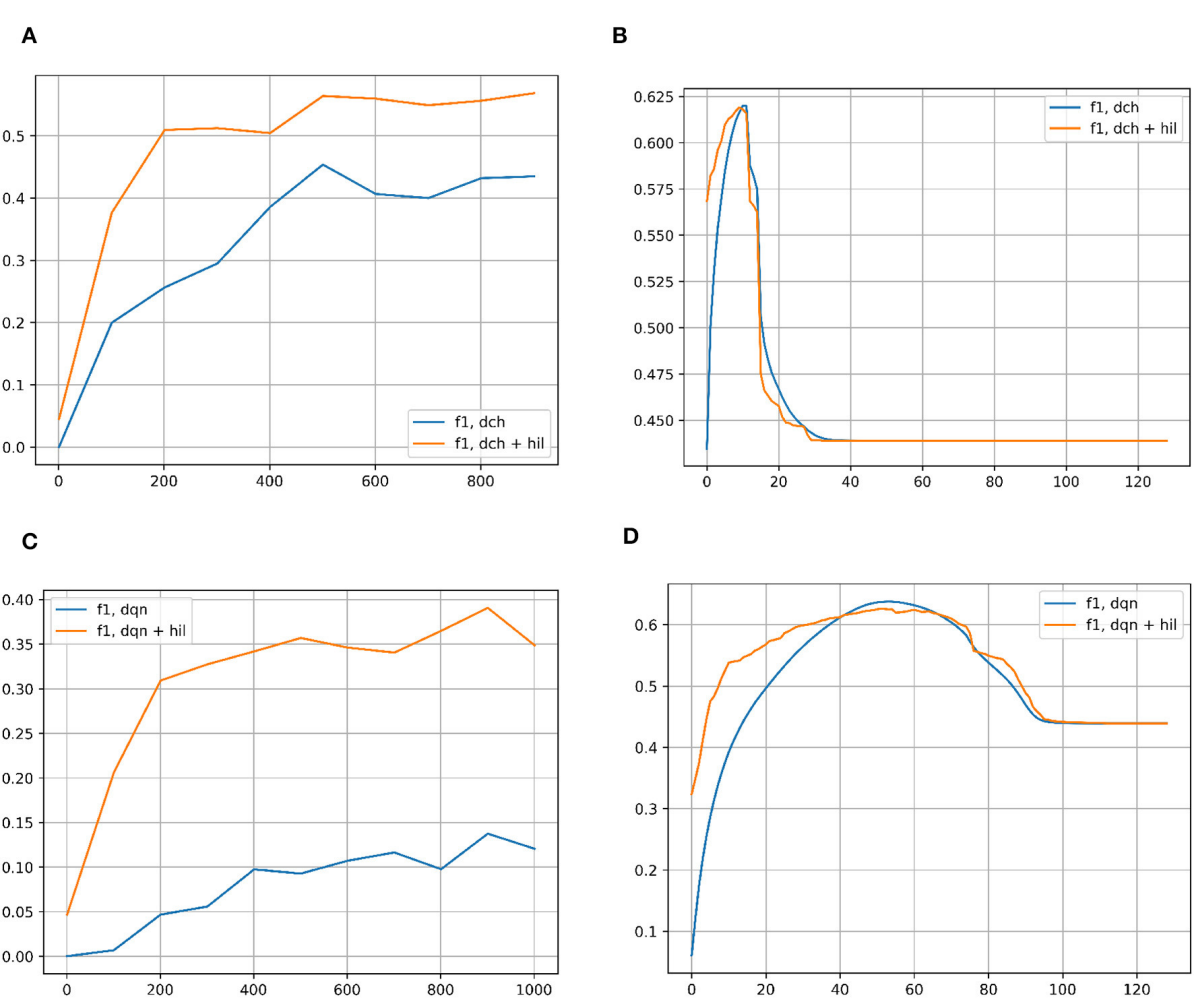
**(A)** F1 score for classification on the NUSWIDE-81 dataset with DCH with and without the HIL, as a function of the number of iterations of training of the DCH network. **(B)** F1 score for classification on the NUSWIDE-81 dataset with DCH with and without the HIL, as a function of the Hamming Distance for classification. The networks are fully trained to the end point shown in subplot **(A)**. **(C)** F1 score for classification on the NUSWIDE-81 dataset with DQN with and without the HIL, as a function of the number of iterations of training of the DQN network. **(D)** F1 score for classification on the NUSWIDE-81 dataset with DQN with and without the HIL, as a function of the Hamming Distance for classification. The networks are fully trained to the end point shown in subplot **(C)**. Baseline networks are shown in blue, while the same network with a HIL appended to the end is shown in yellow. Note that in the left column of subplots, the Hamming Distance for classification is set to 2 for inlier/outlier count. Results for DTQ are omitted for incompatibility with NUWSIDE-81. We largely get the same results in the left column as with CIFAR-10, showing an improvement in performance versus training iterations when an HIL is appended to the end of the baseline network, which adds negligible memory/computation costs. In the right column of results, the HIL differs from CIFAR-10's results in that there is a peak to the performance of the HIL enhanced network. This is likely due to NUSWIDE-81 being designed for the task of web image annotation and retrieval.

Figure 7 compares the F1 scores of each hashing network with and without the HIL on NUSWIDE-81. As with CIFAR-10, the left column shows the performance of each hashing network across iterations of network training. The threshold for Hamming Distance is once again set to 2 bits out of 128 for the baseline networks. For HIL, the distance is proportionally scaled to hyperdimensional lengths in 8,000. Once again, in all cases the HIL greatly improves the F1 score. In the right column, the F1 score is shown for successively more lax Hamming Distances in both methods, retrieving the best match in the Hamming ball of that size. In the case of hyperdimensional vectors, the distances are scaled up to the appropriate values. For each baseline hashing network, there is clearly an optimal Hamming Distance to use, though it is much less pronounced with HIL. In all cases, it is safer to use a smaller Hamming Distance rather than a larger one, except near the optimal values.

### 6.2. Results for the Consensus Architecture

We tested the capability of hyperdimensional computing to fuse the results of different models at the vector-symbolic level. This setup allows to compensate for the shortcomings of the individual models and give a more robust result - a desirable property of hyperdimensional representations. We tested the consensus pipeline on all three hashing networks and on CIFAR-10's full dataset, with fully trained hashing networks and HILs for each. The F1 scored increased to 0.79, ~10% more than the scores any of the models achieved individually with HIL, as seen in Figure 6. This confirms our suspicion that direct fusion at the symbolic level gives far more robust results.

### 6.3. Summary of Experimental Results

Our experiments indicate no performance downside to adding an HIL to an existing, Deep Hash Network. Indeed, it seems that the HIL enables better results with fewer epochs and even improves the F1 score. Furthermore, fusion of multiple networks into a single HIL increased the F1 score greatly above any of the individual networks, even with an HIL. Since each Hash Network formulation differs significantly from each other, one network might be better suited at hashing particular information. We surmise the improvement of performance is because the robustness of the HIL allows each network to naturally contribute its classification to the overall classification decision in a consensus-like fashion.

It should be noted that hyperdimensional computations are very fast. The pyhdc package is designed to perform these computations very efficiently. As a result, the addition of the HIL, in either experiment, is negligible in terms of extra computations and execution time. This is in line with previous results shown in HAP (Mitrokhin et al., 2019), where in a matter of milliseconds the HIL can be trained, retrained from scratch, and even perform classification, on a standard CPU processor. In our results, the HIL also incurred milliseconds of additional runtime. This further indicates that there is virtually no downside to adopting the hyperdimensional approach presented in our architecture.

## 7. Discussion

Hyperdimensional computing has many attractive properties. Our results confirm the notion that hyperdimensional representations can be useful in VSA and symbolic reasoning systems. It is also important to note that hyperdimensional vectors have not yet been effectively used to represent dense RGB images in prior work. This potentially opens up new avenues for combining symbolic reasoning and ML methods. Hyperdimensional representations produced by converting the output of deep hashing networks into symbolic inference structures allows the use of fuzzy logic systems, of which the use of HILs in our experiments are a simple example of. Since HIL structures can be fused across different modalities, this increases the robustness and interpretability of the inference process. We have shown the potential advantages of multi-modal fusion in the HIL by combining three separately trained, differently constructed deep hashing networks without the need of any additional training or oversight, improving the overall result. This is despite the fact that each model is successively more state-of-the-art, meaning that there is no catastrophic loss in integrating newer models into the inference system as more are developed.

Although the results so far are quite interesting and point to a potential future of hyperdimensional computing in the marriage of ML and symbolic reasoning systems, there are still many drawbacks to the approach we have presented. First of all, it would be preferable to use non-hashing (or perhaps even non-supervised) networks to bootstrap our system, as these tend to perform much better than hashing methods. However, this would require the ability to convert embeddings in a more sophisticated neural system into corresponding binary vectors. Special quantization methods may need to be developed to facilitate this in future work in order to fully take advantage of hyperdimensional representations.

It is clear that more work is required to fully integrate hyperdimensional representations into ML systems. Specifically, these need to be more compliant to deep representations of features. There are many avenues of future research that can improve upon these limitations, especially in regard to special conversion between deep features in different modalities, such as text, and images. On the symbolic reasoning side, our results do not produce a full-scale, fully realized symbolic system. For example, Figure 1 would indicate that, given the high likelihood of detection of a dog, the system could reason that there is a high likelihood that what is currently observed likes to bark, has four legs, and loves to wag its tail. However, it is not clear how this linguistic knowledge would be incorporated into the associated hyperdimensional space. One can imagine knowledge graph like structures overlaying the hyperdimensional space, or perhaps more sophisticated structures, but it is not readily clear what the best formulation is.

Furthermore, we must point out some of the drawbacks of using hyperdimensional representations to facilitate a connection between data-driven systems and symbolic reasoning systems:

- We have the necessary requirement that data-driven systems can be readily converted into long binary vectors. This is a severe restriction, as most state-of-the-art methods naturally use real-valued computations. Most neural methods produce samples on complex manifolds that may be difficult to effectively map to hyperdimensional vectors. Thus, there is a need for a general technique to project real-valued embeddings from data-driven systems to binary spaces. As a result, real-value hyperdimensional vectors may be better suited to certain tasks (Summers-Stay et al., 2018; Sutor et al., 2019).
- Along the same lines, many modern-day symbolic reasoning systems also rely on real-value computations or representations, especially when data driven. New methods would have to be developed to work with more sophisticated systems.
- While hyperdimensional vector representations of different modalities can be embedded effectively into a common space, they may also require a nearest neighbor lookup when looking for similar, known concepts. This may become expensive when the hyperdimensional space contain many concepts. In order to maintain that data of a particular modality is closer to other examples of that modality, it may be necessary to adopt an approach that facilitates this, such as in Sutor et al. (2018).

## Data Availability Statement

The datasets analyzed for this study can be found on the DeepHash project page (https://github.com/thulab/DeepHash). The *pyhdc* library code used in this work can be found in https://github.com/ncos/pyhdc.

## Author Contributions

AM contributed to the experiments, evaluations, plots, and text of the manuscript. PS contributed to experiments, the text of the manuscript, and illustrations. DS-S, CF, and YA contributed to the text of the manuscript. All authors contributed to the conceptual ideas at the heart of this research.

## Conflict of Interest

The authors declare that the research was conducted in the absence of any commercial or financial relationships that could be construed as a potential conflict of interest.

## Footnotes

^1^Code repository available on GitHub at https://github.com/thulab/DeepHash.

^2^Code repository available on GitHub at https://github.com/ncos/pyhdc.

## References

[B1] Cao Y. Long M. Liu B. Wang J. (2018). Deep cauchy hashing for hamming space retrieval, in Proceedings of the IEEE Conference on Computer Vision and Pattern Recognition (Salt Lake City, UT), 1229–1237. 10.1109/CVPR.2018.00134

[B2] Cao Y. Long M. Wang J. Liu S. (2017). Deep visual-semantic quantization for efficient image retrieval, in Proceedings of the IEEE Conference on Computer Vision and Pattern Recognition (Honolulu, HI), 1328–1337. 10.1109/CVPR.2017.104

[B3] Cao Y. Long M. Wang J. Zhu H. Wen Q. (2016). Deep quantization network for efficient image retrieval, in Thirtieth AAAI Conference on Artificial Intelligence (Phoenix, AZ).

[B4] Chua T.-S. Tang J. Hong R. Li H. Luo Z. Zheng Y. (2009). NUS-wide: a real-world web image database from National University of Singapore, in Proceedings of the ACM International Conference on Image and Video Retrieval (Santorini), 1–9. 10.1145/1646396.1646452

[B5] Deng J. Dong W. Socher R. Li L.-J. Li K. Fei-Fei L. (2009). Imagenet: a large-scale hierarchical image database, in 2009 IEEE Conference on Computer Vision and Pattern Recognition (Miami, FL: IEEE), 248–255. 10.1109/CVPR.2009.5206848

[B6] Imani M. Huang C. Kong D. Rosing T. (2018). Hierarchical hyperdimensional computing for energy efficient classification, in 2018 55th ACM/ESDA/IEEE Design Automation Conference (DAC) (San Francisco, CA: IEEE), 1–6. 10.1109/DAC.2018.8465708

[B7] Imani M. Kong D. Rahimi A. Rosing T. (2017). Voicehd: hyperdimensional computing for efficient speech recognition, in 2017 IEEE International Conference on Rebooting Computing (ICRC) (McLean, VA: IEEE), 1–8. 10.1109/ICRC.2017.8123650

[B8] Jimenez L. O. Morales-Morell A. Creus A. (1999). Classification of hyperdimensional data based on feature and decision fusion approaches using projection pursuit, majority voting, and neural networks. IEEE Trans. Geosci. Rem. Sens. 37, 1360–1366. 10.1109/36.763300

[B9] Kanerva P. (2009). Hyperdimensional computing: an introduction to computing in distributed representation with high-dimensional random vectors. Cogn. Comput. 1, 139–159. 10.1007/s12559-009-9009-8

[B10] Kleyko D. Rahimi A. Rachkovskij D. A. Osipov E. Rabaey J. M. (2018). Classification and recall with binary hyperdimensional computing: tradeoffs in choice of density and mapping characteristics. IEEE Trans. Neural Netw. Learn. Syst. 29, 5880–5898. 10.1109/TNNLS.2018.2814400 29993669

[B11] Krizhevsky A. Hinton G. (2009). Learning Multiple Layers of Features From Tiny Images. The University of Toronto.

[B12] Krizhevsky A. Sutskever I. Hinton G. E. (2012). Imagenet classification with deep convolutional neural networks, in Advances in Neural Information Processing Systems (Lake Tahoe), 1097–1105.

[B13] Liu B. Cao Y. Long M. Wang J. Wang J. (2018). Deep triplet quantization, in Proceedings of the 26th ACM International Conference on Multimedia (Seoul), 755–763. 10.1145/3240508.3240516

[B14] Mikolov T. Chen K. Corrado G. Dean J. (2013). Efficient Estimation of Word Representations in Vector Space. Scottsdale, AZ: International Conference on Learning Representations (ICLR).

[B15] Mitrokhin A. Sutor P. Fermüller C. Aloimonos Y. (2019). Learning sensorimotor control with neuromorphic sensors: Toward hyperdimensional active perception. Sci. Robot. 4:eaaw6736 10.1126/scirobotics.aaw6736 33137724

[B16] Moon S. Berster B.-T. Xu H. Cohen T. (2013). Word sense disambiguation of clinical abbreviations with hyperdimensional computing, in AMIA Annual Symposium Proceedings, Vol. 2013 (Washington, DC: American Medical Informatics Association), 1007. 24551390 PMC3900125

[B17] Pennington J. Socher R. Manning C. D. (2014). Glove: global vectors for word representation, in Proceedings of the 2014 Conference on Empirical Methods in Natural Language Processing (EMNLP) (Doha), 1532–1543. 10.3115/v1/D14-1162

[B18] Rahimi A. Datta S. Kleyko D. Frady E. P. Olshausen B. Kanerva P. (2017). High-dimensional computing as a nanoscalable paradigm. IEEE Trans. Circuits Syst. I Reg. Pap. 64, 2508–2521. 10.1109/TCSI.2017.2705051

[B19] Rahimi A. Kanerva P. Rabaey J. M. (2016). A robust and energy-efficient classifier using brain-inspired hyperdimensional computing, in Proceedings of the 2016 International Symposium on Low Power Electronics and Design (San Francisco, CA), 64–69. 10.1145/2934583.2934624

[B20] Summers-Stay D. Sutor P. Li D. (2018). Representing sets as summed semantic vectors. Biol. Inspired Cogn. Archit. 25, 113–118. 10.1016/j.bica.2018.07.002

[B21] Sutor P. Aloimonos Y. Fermuller C. Summers-Stay D. (2019). Metaconcepts: isolating context in word embeddings, in 2019 IEEE Conference on Multimedia Information Processing and Retrieval (MIPR) (San Jose, CA: IEEE), 544–549. 10.1109/MIPR.2019.00110

[B22] Sutor P. Summers-Stay D. Aloimonos Y. (2018). A computational theory for life-long learning of semantics, in International Conference on Artificial General Intelligence (Prague: Springer), 217–226. 10.1007/978-3-319-97676-1_21

[B23] Yilmaz O. (2015). Symbolic computation using cellular automata-based hyperdimensional computing. Neural Comput. 27, 2661–2692. 10.1162/NECO_a_00787 26496041

[B24] Zhu H. Long M. Wang J. Cao Y. (2016). Deep hashing network for efficient similarity retrieval, in Thirtieth AAAI Conference on Artificial Intelligence (Phoenix, AZ).

